# Determinants of QuantiFERON Plus-diagnosed tuberculosis infection in adult Ugandan TB contacts: A cross-sectional study

**DOI:** 10.1371/journal.pone.0281559

**Published:** 2023-03-27

**Authors:** Jonathan Mayito, Adrian R. Martineau, Divya Tiwari, Lydia Nakiyingi, David P. Kateete, Stephen T. Reece, Irene Andia Biraro

**Affiliations:** 1 Department of Immunology and Molecular Biology, School of Biomedical Sciences, Makerere University College of Health Sciences, Kampala, Uganda; 2 Infectious Diseases Institute, Makerere University College of Health Sciences, Kampala, Uganda; 3 Centre for Immunobiology, Blizard Institute, Barts and The London School of Medicine and Dentistry, Queen Mary University of London, London, United Kingdom; 4 Department of Internal Medicine, Makerere University College of Health Sciences, Kampala, Uganda; 5 School of Clinical Medicine, University of Cambridge, Cambridge, United Kingdom; 6 MRC/UVRI & LSHTM Uganda Research Unit, Entebbe, Uganda; Aurum Institute, SOUTH AFRICA

## Abstract

**Background:**

The tuberculin skin test is commonly used to diagnose latent tuberculosis infection (LTBI) in resource-limited settings, but its specificity is limited by factors including cross-reactivity with BCG vaccine and environmental mycobacteria. Interferon-gamma release assays (IGRA) overcome this problem by detecting *M*. *tuberculosis* complex-specific responses, but studies to determine risk factors for IGRA-positivity in high TB burden settings are lacking.

**Methods:**

We conducted a cross-sectional study to determine factors associated with a positive IGRA by employing the QuantiFERON-TB^®^ Gold-plus (QFT Plus) assay in a cohort of asymptomatic adult TB contacts in Kampala, Uganda. Multivariate logistic regression analysis with forward stepwise logit function was employed to identify independent correlates of QFT Plus-positivity.

**Results:**

Of the 202 participants enrolled, 129/202 (64%) were female, 173/202 (86%) had a BCG scar, and 67/202 (33%) were HIV-infected. Overall, 105/192 (54%, 95% CI 0.48–0.62) participants had a positive QFT Plus result. Increased risk of QFT-Plus positivity was independently associated with casual employment/unemployment vs. non-casual employment (adjusted odds ratio (aOR) 2.18, 95% CI 1.01–4.72), a family vs. non-family relation to the index patient (aOR 2.87, 95% CI 1.33–6.18), living in the same vs. a different house as the index (aOR 3.05, 95% CI 1.28–7.29), a higher body mass index (BMI) (aOR per additional kg/m^2^ 1.09, 95% CI 1.00–1.18) and tobacco smoking vs. not (aOR 2.94, 95% CI 1.00–8.60). HIV infection was not associated with QFT-Plus positivity (aOR 0.91, 95% CI 0.42–1.96).

**Conclusion:**

Interferon Gamma Release Assay positivity in this study population was lower than previously estimated. Tobacco smoking and BMI were determinants of IGRA positivity that were previously unappreciated.

## Background

Tuberculosis (TB) ranks second after Covid-19 among the leading infectious causes of death. In 2020, it was responsible for 1.3 million and 214, 000 deaths among HIV-uninfected and HIV-infected people respectively [1]. Estimates indicate that 25% of the global population has latent TB and holds a 10% life time risk of reactivation to active disease, usually within 2–5 years following exposure [2]. Latent TB infection (LTBI) is diagnosed using immunological tests, due to lack of a gold standard microbiologic test, with the interferon gamma release assay (IGRA) being preferred to Tuberculin Skin Test (TST) because of its higher specificity [3]. This arises as a consequence of the IGRA’s reduced potential for cross-reactivity with antigens from *Mycobacterium bovis* and environmental mycobacteria [4].

However, IGRAs have a low positive predictive value (PPV), 2.7%, for risk of reactivation to active TB [5]. Therefore the individuals to whom the test is applied should be those at a higher risk of reactivation, who are more likely to benefit from preventive therapy [5]. The TB2 antigen in QuantiFERON-TB Gold PLUS (QFT-PLUS) stimulates both CD4+ and CD8+ T cells. Detection of CD8+ T cells responses improves the PPV to 5.7%, which reflects increased responses early after exposure when risk for TB progression is highest [6, 7]. Targeting individuals at high risk of reactivation is critical as a stronger protective effect from isoniazid preventive therapy was observed in individuals with a positive TST than in those with a negative TST [8]. Therefore, provision of TB preventative therapy based on symptom-based screening alone, a practice used in most high burden settings, will expose many individuals at low risk of TB progression to potentially toxic drugs. Specificity of symptom-based screening may be improved by integrating determinants of IGRA positivity.

These determinants have largely been evaluated in low-burden settings and are likely distinct from those present in high-burden settings. Male gender, intravenous drug use, CD4 count of 200–349 per μl, and a past history or exposure to *M*. *tuberculosis* were associated with a positive IGRA among refugees in Australia [9]. Likewise, older age, Indian ethnicity, being foreign-born, primary or lesser education, and alcohol consumption more than once a week, were associated with a positive IGRA in Singaporean adults aged 18–79 years [10]. Diabetes and pregnancy, conditions associated with reduced immunity, are associated with lower IGRA positivity while the rate of indeterminate IGRA results is increased in pregnancy but not in diabetes [11–13]. On the other hand, alcohol use, advanced age and history of pulmonary TB disease were risk factors for indeterminate IGRA results in a Portuguese national TB survey [14]. Also, young age, malarial infection, iron deficiency anemia and helminth infestation were associated with indeterminate IGRA results [15] while advanced age (> 60 years) and low lymphocyte counts, including CD4 count < 200, were associated with false negative IGRA results [16].

The determinants of TST and IGRA positivity differ with advancing age being the common factor independently associated with the two [16, 17]. The difference might be due to IGRA being based on the region of difference-1 (RD-1) *M*. *tuberculosis* specific antigens that make it non-cross reactive with BCG and environmental mycobacteria antigens [18]. Lou et al found that male sex, dental surgery degree studies and close contact with index patient were independently associated with TST positivity among Ugandan medical students [19]. In Morocco, a study by Sabri et al. 2019 showed that male sex, age groups 34–45 years—more so 45–60 years, family history of TB as well as working in a pulmonary unit were strong predictors of TST positivity [17]. Birth in a high-risk country and male gender were also independently associated with a positive TST result [20] while contact with an HIV negative index patient as opposed to an HIV positive one was associated with higher odds of a positive TST [21]. A higher baseline TST induration, older age (>15 years), and a higher epidemiological risk score were independently associated with TST conversion and time to TST conversion in a Ugandan cohort of household contacts of index TB patients. The majority of TST conversions happened within six months, suggesting that TST can be used as a biomarker to detect people who are at risk of developing active TB [22]. On the other hand, HIV infection alters the reactivity of TST because it impairs the cell-mediated immunity that forms the basis of the test [23]. Consequently, the absolute CD4 and total lymphocyte counts have a positive correlation with the TST reactivity [24] and a lower cut of TST reactivity of 5 mm rather than 10 mm or higher for HIV infection is adopted. The TST reactivity is also reduced in other immune suppressive conditions like pregnancy [25], diabetes, renal disease, or organ transplant [26].

We therefore sought to evaluate the determinants of IGRA positivity in asymptomatic close contacts of index pulmonary TB patients in a high burden setting. Close contacts are recently exposed and therefore at a higher risk of infection and progression to active TB. In addition, risk factors associated with recent infection may differ from those of remote infection.

## Materials and methods

Ethical approval was obtained from Makerere University School of Biomedical Sciences Institutional Review Board, reference number SBS 595. Informed consent was obtained from all participants before any study related procedures were conducted. This was a cross sectional study carried out between June 2019 and June 2020, among close contacts of index pulmonary TB patients aged 18 years or more with a negative TB symptom screen, a normal CXR and no prior treatment with TB drugs. Participants were recruited from the Infectious Diseases Institute, Mulago National Referral Hospital and Kampala City Council Authority TB clinics. A close contact was taken as an individual who spent a night or frequent or extended daytime periods with an index pulmonary TB patient during the 3 months before they initiated treatment [27]. Identification of bacteriologically confirmed index pulmonary TB patients was carried out in the above TB clinics and they were requested for permission for their contacts to participate in the study.

The close contacts were invited to give written consent to participate in the study after being briefed about the study. Consenting participants gave social demographic information and a detailed history of contact with the index pulmonary TB patient including: number of index pulmonary TB patients the close contact had contact with, relationship with the index patient, proximity of contact, intensity of contact and duration the index patient had coughed prior to initiating treatment. Also, information was collected on bacillary load in the index patient’s sputum results, smoking status, BCG immunization status and for HIV positive participants, last viral load results. Formal employment, private business, casual laborer, peasant, and unemployed were the subcategories assessed for occupation. In order to have higher-powered sample numbers, these were re-categorized according to the risk they pose for *M*. *tuberculosis* infection, i.e., congestion and other risky working and living conditions. Because they pose comparable hazards, formal employment and private business were classed as non-casual employment, while the rest was categorized as casual employment or unemployment. Recruited participants underwent a physical examination including: measurement of weight, height, and temperature, as well as examination for peripheral lymphadenopathy, chest auscultation, abdominal palpation for masses, and skeletal system examination for joint swelling and vertebral column gibus. Participants then donated 5 ml of blood, of which 4 ml were used for IGRA testing and 1 ml for HIV testing. The IGRA samples were analyzed using QuantiFERON-TB^®^ Gold-plus (QFTPlus) according to the manufacture’s standard operating procedures while HIV testing was done according to the national testing algorithm [28].

Sample size Sample size was predicated on the requirement for 10–20 participants per independent variable included in regression analyses [29]: a total of 202 participants were recruited to investigate associations for 16 independent variables.

## Statistical analysis

In descriptive analysis, continuous variables were reported as means with the standard deviation (SD) while categorical variables were reported as proportions in terms of frequencies and percentages. In inferential analysis, a random effect logistic regression analysis was used to determine factors associated with a positive IGRA test. Univariate logistic regression models were used to obtain unadjusted odds ratios for all characteristics potentially associated with a positive IGRA. Multivariate logistic regression analysis with forward stepwise logit function was done with variables included as a priori based on biological plausibility with IGRA positivity. Adjusted odds ratio were reported with 95% confidence intervals (95% CI). Data was analyzed using Stata/IC 15.0, StataCorp LLC Texas USA. For all comparisons, a two-tailed P-value < 0.05 was considered significant.

## Results

Participant socio-demographic characteristics are shown in Table 1. Of the 202 participants enrolled, 129/202 (64%) were female, 173/202 (86%) had a BCG scar, and 67/202 (33%) were from an HIV positive cohort. The average age was 31 (SD: 10) years, while 130/202 (64%) had a BMI <25 kg/m^2^. The majority, were related to [142/202 (70%)], lived in the same house as [150/202 (74%)], or spent more than half of the day [156/202 (77%)] with the index patient. Up to 109/202 (54%) participants had attained post-primary education, 151/202 (75%) were in casual employment or unemployed while only 32/202 (16%) were tobacco smokers. Casual employment here referred to an informal employment, which was physical in nature and occurred in a congested work environment.

**Table 1:** Participant characteristics (n = 202).

Independent variable	Category	Number (%)
**Age, yrs**.	18–29.9	103 (51)
	30–49.9	85 (42)
	>50	14 (7)
**Sex**	F	129 (64)
	M	73 (36)
**Education**	Post-primary	109 (54)
	Primary or less	93 (46)
**1Occupation**	Non-casual employment	51 (25)
	casual employment or unemployed	151 (75)
**BMI, kg/m** ^**2**^	Underweight (< 18.5)	16 (8)
	Normal weight (18.5–24.9)	130 (64)
	Overweight (> 25)	56 (28)
2**Index cases exposures (no. index cases)**	1	164 (81)
	2 or more	38 (19)
**Place of contact with index case**	Non-household contact	23 (11)
	Household contact	179 (87)
**Relation with index**	Non-family relation	60 (30)
	Family relation -	142 (70)
**Exposure intensity to index case**	<50% of day	45 (23)
	>50% of day	156 (77)
**Proximity to index case**	Different houses	52 (26)
	Same house	150 (74)
**Tobacco smoking**	Not a smoker	170 (84)
	Current smoker	32 (16)
**HIV status**	Uninfected	135 (67)
	Infected	67 (33)
**QFT status**	Negative	87 (43)
	Positive	105 (52)
	Intermediates	10 (5)
**BCG status**	No scar	28 (14)
	Scar present	173 (86)
**Viral load suppression (cut off 1000 copies/mL)**	Suppressed viral load	61 (91)
	Non-suppressed viral load	6 (9)

1: Non-casual employment consisted of formal employment and private business while casual employment or unemployment included casual laborer, peasant, and unemployed. Casual employment here referred to an informal employment, which was physical in nature and occurred in a congested work environment, 2: Each index cases was enumerated once even if the close contact had numerous contacts with the index case, say in the case of a household setting.

### Prevalence of IGRA positivity and associated factors

**Fig 1:**
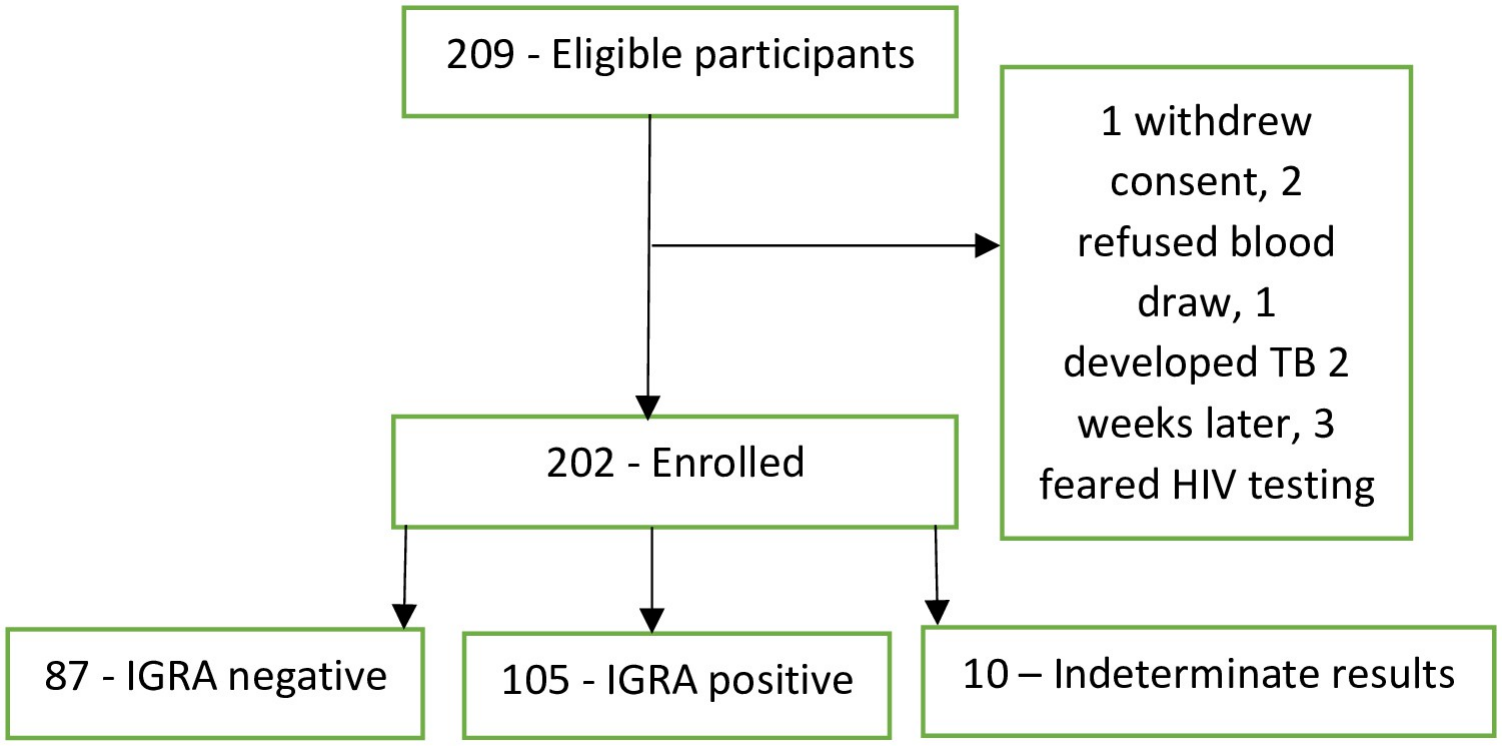
Subject recruitment and IGRA outcomes.

After excluding 10/202 (5%) participants with indeterminate results, 105/192 (54%, 95% CI 0.48–0.62) participants had a positive QFT-Plus result (Fig 1).

Fifty five percent (36/65) of HIV positive participants had a positive QFT-Plus result compared to 69/127 (54%) of HIV negative participants. More participants aged 50 years and above, 9/14 (64%), were QFT-Plus positive compared to the younger age groups as were tobacco smokers, 20/31 (65%), compared to non-smokers, 85/161 (53%).

Using bivariate logistic regression, five factors were independently associated with an increased risk of a positive QFT-Plus result: unemployment or in casual employment vs. non-casual employment (Odds Ratio (OR): 2.02, 95% CI 1.04–3.91), household vs. non-household as place of contact (OR: 2.92, 95% CI 1.13–7.52), family vs. non-family relation to the index patient (OR: 2.96, 95% CI 1.56–5.61), living in the same vs. different houses as the index patient (OR: 2.24, 95% CI 1.16–4.32), and a higher BMI (OR per kg/m^2^: 1.08, 95% CI 1.00–1.16). Using multivariate logistic regression, in casual employment or unemployment vs. non-casual employment (adjusted OR [aOR]: 2.18, 95% CI 1.01–4.72), family vs. non-family relation to the index patient (aOR: 2.87 CI 1.336.18), living in the same house vs. different houses as the index patient (aOR: 3.05, 95% CI 1.28–7.29), and a high BMI (aOR per kg/m^2^: 1.09, 95% CI 1.00–1.18) remained independently associated with an increased risk of a positive QFT-Plus result whereas household vs. non-household location of index case contact was no longer significant. In addition, smoking vs. not smoking tobacco (aOR: 2.94 CI 1.00–8.60) was also independently associated with an increased risk of a positive QFT-Plus result (Table 2).

**Table 2:** Factors associated with IGRA positivity.

Independent variable	Category	Proportion QFT-positive n/N (%)	Univariate analysis		Multivariable analysis	
			Unadjusted odds ratio	P-value	Adjusted odds ratio^1^	P-value
**Age, yrs**.	18–29.9	56/99 (57)	Ref			
	30–49.9	40/79 (51)	0.79 [0.43–1.43]	0.430	1.00 [0.97–1.04]	0.983
	>50	9/14 (64)	1.38 [0.43–4.42]	0.586		
**Sex**	Female	68/124 (55)	Ref		Ref	
	Male	37/68 (54)	0.98 [0.54–1.78]	0.955	0.93 [0.45–1.94]	0.854
**Education**	Post-primary	54/104 (52)	Ref		Ref	
	Primary or less	51/88 (58)	1.28 [0.72–2.26]	0.403	0.96 [0.47–1.95]	0.906
**Occupation**	Non-casual employment	20/48 (42)	Ref		Ref	
	Casual employment or unemployed	85/144 (59)	2.02 [1.04–3.91]	0.038	2.18 [1.01–4.72]	0.047
**BMI, kg/m** ^**2**^			1.08 [1.00–1.16]	0.040	1.09 [1.00–1.18]	0.049
**Index cases participant was exposed**	1	84/155 (54)	Ref			
	2 or more	21/37 (57)	1.11 [0.54–2.29]	0.778	1.09 [0.47–2.49]	0.845
**Place of contact with index case**	Non-household contact	7/22 (32)	Ref		Ref	
	Household contact	98/170 (58)	2.92 [1.13–7.52]	0.027	2.50 [0.75–8.38]	0.138
**Relation with index**	Non-family relation	21/58 (36)	Ref		Ref	
	Family relation	84/134 (63)	2.96 [1.56–5.61]	0.001	2.87 [1.336.18]	0.007
**Exposure intensity to index case**	<50% of day	24/45 (53)	Ref		Ref	
	>50% of day	81/147 (55)	1.07 [0.55–2.10]	0.835	0.72 [0.31–1.69]	0.451
**Proximity to index case**	Different houses	20/50 (40)	Ref		Ref	
	Same house	85/142 (60)	2.24 [1.16–4.32]	0.016	3.05 [1.28–7.29]	0.012
**Tobacco smoking**	Not a smoker	85/161 (53)	Ref		Ref	
	Current smoker	20/31 (65)	1.63 [0.73–3.61]	0.233	2.94 [1.00–8.60]	0.050
**HIV status**	Uninfected	69/127 (54)	Ref		Ref	
	Infected	36/65 (55)	1.04 [0.57–1.90]	0.890	0.91 [0.42–1.96]	0.810
**BCG status**	No scar	13/27 (48)	Ref		Ref	
	Scar present	92/165 (56)	1.36 [0.60–3.066]	0.463	1.07 [0.39–2.90]	0.898
1 **Sputum bacillary load, index case**	Low	22/41 (54)	Ref			
	High	74/133 (56)	1.08 [0.54–2.19]	0.824	N/A	N/A
	Not known	9/18 (50)	0.86 [0.54–2.19]	0.796	N/A	N/A
2 **Cough duration, index case**	< 1 month	16/29 (55)	Ref		N/A	N/A
	≥ 1 month	70/129 (54)	0.96 [0.43–2.17]	0.929	N/A	N/A
	Not known	19/34 (56)	1.03 [0.38–2.79]	0.955	N/A	N/A

Index case sputum bacillary load (1) and cough duration (2) were not significant in the univariate analysis and did not fit in the final multivariable model. N/A—Not applicable.

**Fig 2:**
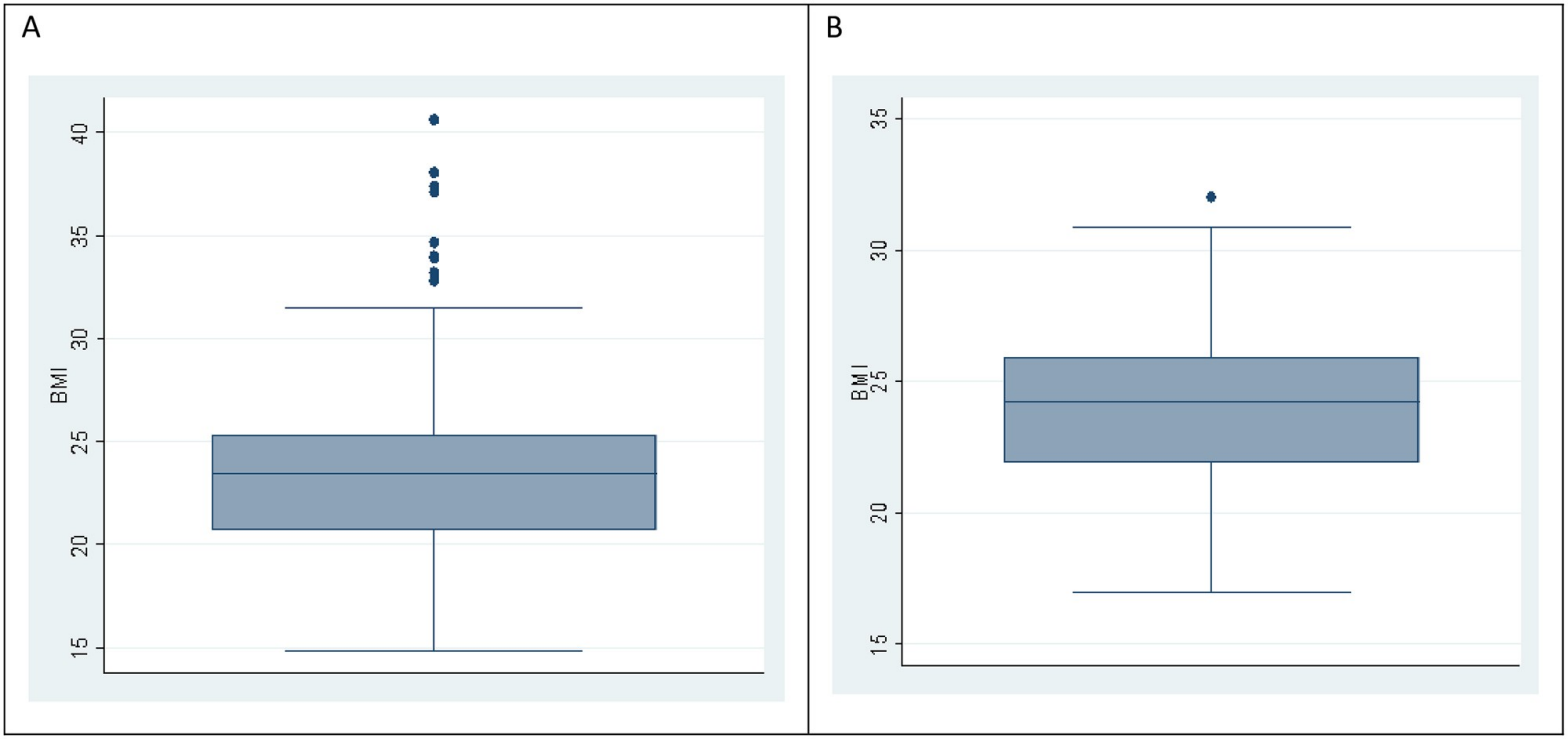
Distribution boxplots of BMI for participants with positive QFTPlus (A) and negative QFTPlus (B) results.

The distribution of BMI for participants with a positive QFTPlus result was slightly positively skewed (Fig 2) compared to that of participants with a negative QFTPlus results which was normally distributed.

## Discussion

We found a 54% prevalence of QFT-Plus positivity, which did not differ between HIV positive and HIV negative participants. Tobacco smoking and BMI are hitherto unrecognized factors associated with risk of a positive QFT-Plus result. Family relation, living in the same house as the index patient, and a casual employment or unemployment were the other factors associated with a positive QFT-Plus result.

The prevalence of IGRA positivity in our study was less than that reported by Biraro et al (65%) in the same setting [30]. In both studies, the study population was asymptomatic close contacts of index pulmonary TB patients; however, Biraro et al included children less than 18 years. Children, particularly those less than 5 years carry a higher risk for TB infection and disease because of immature immune system [31] but the prevalence is usually higher in adults because of longer periods of exposure. Further, Biraro et al employed QFT-Gold In-Tube compared to QFT-Plus employed in this study. The QFT-Plus is considered more sensitive than QFT-Gold In-Tube because of the inclusion of the TB2 antigen which also stimulates CD8+ T cells in addition to CD4+ T cells [32]. The prevalence did not differ between HIV positive and HIV negative participants, despite the heightened risk of TB infection for HIV positive individuals following exposure and for reactivation to active disease [33]. This was also contrary to the observation that IGRAs have sub-optimal performance in immunosuppressive conditions like HIV, particularly in those with reduced CD4 counts [34]. The good performance reflected here among the HIV positive could have resulted from majority of the HIV positive participants having a suppressed viral load, likening their immune response to that of the HIV negatives participants. We also acknowledge a higher HIV positivity among this cohort compared to other TB contacts cohort or to the general population. The HIV prevalence in household contacts was estimated at 13% [35] and that in the general population at 5.8% [36].

Close contact with an index TB patient is a strong risk factor for TB infection [37], and first degree relatives are particularly at a heightened risk [38]. In this study, a family relation and living in the same house as the index case were associated with a significant risk for a positive QFT-Plus result. This was similar to findings by Jung et al study among household contacts in South Korea, where living in the same room as the index patient was associated with a positive IGRA [39], and findings by Hermann et al that family relations including a diseased sibling or sexual partner were associated with an increased risk for positive IGRA [40]. Using IGRA as either a categorical or quantitative trait, sleeping in the same room as a symptomatic index patient or simply contact within household were associated with IGRA-positivity in Colombia [41]. The household risk of TB transmission was dependent on the level of crowding within the households [42]. On the contrary, Shanaube et al didn’t find any association between sleeping in close proximity to the index patient and risk of TB infection [43]. Further, other studies have suggested that in high burden settings, the risk of transmission in the household and the community may not differ [43, 44]. No clear explanation has been put forward for this observation but it may be due to multiple exposures from unidentified index patients in the community.

Nutritional status, for which BMI may be a marker, can influence cell-mediated immunity and low BMI is associated with higher TB incidence [45, 46]. Further, a higher BMI has been shown to be protective against incident TB but the effect was not apparent with BMI >30 kg/m^2^ [47]. Furthermore, cachexia that can occur during active TB results from hypothalamic hormone regulation by peptide YY, grehlin and resistin [48]. In this study, for every 1 Kg/M^2^ increase in BMI, there was an associated 9% increase in risk of a positive QFT-Plus result. Similar to our finding, Zhang et al showed that a BMI of > 28 Kg/M^2^ was a risk factor for a positive IGRA and the latent TB prevalence increased from 18.5% in underweight to 23.7% in obese participants [49]. Adipose tissue has previously been shown to harbor *M*.*tb* DNA during latent TB infection, with *M*.*tb* gaining entry into adipocytes through scavenger receptors and accumulating in cytoplasmic lipid droplets to become predominantly dormant in mature adipocytes [50]. Adiposity is associated with metabolic disturbances which result in defective innate and adaptive immune responses that might aid susceptibility to infections and reactivation of latent disease [51], including TB. The immune defects include reductions in leucocyte numbers, phagocytosis, oxidative burst and proliferative capacity following antigen stimulation [52]. These immune defects are mediated by immunomodulatory adipokines including leptin, adiponectin and pro-inflammatory cytokines: TNFα, IL-6 and IL-1β [52].

Tobacco smoking is associated with an increased risk for *M*. *tuberculosis* infection and progression to active disease. Exposure to tobacco smoke damages airway ciliary function and suppresses cytotoxic activity of NK cells and T cell function [53, 54]. For instance, in India, up to 40% of TB infections were attributed to smoking [55] while in a study in Taiwan quitting smoking was associated with a more than 65% decrease in mortality [56]. The World Health Organization estimates that 7% of the TB cases in 2020 were attributable to smoking and lists it among the top five risk factors for TB that should be targeted for TB control through the 5A’s and 5R’s models, to ready and motivate patients for quitting smoking [57].

Risk of *M*. *tuberculosis* infection in occupations other than healthcare is understudied [58]. Manual jobs occur in environments likely to promote transmission of TB. For instance, dusty work environments such as mines, stony quarries, building sites, charcoal stores or other exposure to silica are associated with *M*. *tuberculosis* infection [59]. Manual workers are also migratory and more likely to work in congested work places, increasing their risk for exposure to *M*. *tuberculosis*. Smoking, alcohol and HIV, which independently are risk factors for *M*. *tuberculosis* infection and disease, are all more prevalent among manual laborers [60]. On the other hand, having no job is linked to a low socio-economic status, which is characterized by poor living conditions including crowding and poor ventilations. These conditions are associated with a heightened risk for TB transmission [38].

The strengths of our study included the use of IGRA for which immuno-reactivity, unlike TST, is constrained to *M*. *tuberculosis* complex-specific antigens, and in particular QFT-Plus, which contains the TB2 antigen that stimulates CD8+ T cells in addition to the CD4+ T cells. The CD8+ T cells responses are more associated with subclinical and active TB than in latent TB [61]. Secondly, we evaluated a large range of risk factors, which strengthened the study’s ability to test for confounders for IGRA positivity. The study also had some limitations. The cross sectional design employed does not indicate a causal relationship between IGRA positivity and the studied factors. Because the exposure and outcome are measured at the same time, the temporal relationship between the two could not be established. Another major limitation was the assumption that contacts in the community could not have been responsible for the *M*. *tuberculosis* infection in the study participants. This could have led to a higher estimate of risk of *M*. *tuberculosis* infection related to close contact. However, indoor exposure is a stronger risk factor for *M*. *tuberculosis* infection than outdoor exposure hence infections were more likely to be from the indoor exposure or close contacts. Secondly, we used CXR as the additional measure to exclude active TB, which is inferior to other digital imaging techniques like the computed tomography scan. Thirdly, we employed a diagnostic test which reflects immune sensitization that may lack specificity as compared to molecular biomarkers like the detection of *M*.*tb* DNA in CD34+ peripheral blood mononuclear cells [62] or transcriptional signatures for incipient TB [63], which may have higher PPV. Lastly, our study did not quantify the intensity of smoking in terms of pack years. As a result, the study did not comprehensively evaluate the risk of *M*. *tuberculosis* infection posed by smoking.

## Conclusion

Interferon Gamma Release Assay positivity in this study population was lower than previously estimated. Tobacco smoking and BMI were determinants of IGRA positivity that were previously unappreciated.
